# Shaping the past: how donors influenced Becker Library's rare book collections

**DOI:** 10.5195/jmla.2022.1551

**Published:** 2022-10-01

**Authors:** Elisabeth Brander

**Affiliations:** 1 ebrander@wustl.edu, Rare Book Librarian, Bernard Becker Medical Library, Washington University in St. Louis School of Medicine, St. Louis, MO.

**Keywords:** Rare books, special collections, history of medicine

## Abstract

Rare book collections do not form in a vacuum; they are shaped by the individuals who assemble and curate them. This is certainly the case with the rare book holdings of Becker Medical Library at Washington University in St. Louis School of Medicine. This paper examines some of the most significant donors to Becker's rare book collections in order to explore how these collections are a reflection of the interests and priorities of the physicians who assembled them, and also raises the issue of how the makeup of these collections create a Western-focused narrative regarding the history of medicine.

## INTRODUCTION

One of the most outstanding resources held at the Bernard Becker Medical Library at Washington University in St. Louis is its collection of rare books. With volumes dating from the late fifteenth to mid-twentieth centuries, the collection covers more than five hundred years of medical history and contains titles by some of the most prominent names in the history of medicine, including Hippocrates, Andreas Vesalius, William Harvey, and others. This is certainly an impressive collection; however, it only represents a fraction of the history of medicine. Becker's collections are skewed toward European and North American medicine, while other medical traditions are barely represented. This imbalance results from the collecting interests and priorities of the donors whose personal collections now form the institutional collection; however, it also gives the impression that the history of medicine is primarily that of Western medicine rather than a global discipline with many interconnecting traditions.

The question of what makes a rare book in the first place is highly subjective and dependent on a series of human decisions. In his monograph on the development of rare books as a concept, book historian Bruce McKittrick argues that the most significant of these are made by owners, and that the history of rare books is indelibly entwined with the history of ownership. Owners decided which of their old books were kept and which were cast aside, and those books that were regarded as valuable enough to keep, or to seek out and acquire, gradually became tied to the idea of cultural heritage [8]. As noted by McKitterick, early printed books—especially incunabula, or books printed prior to 1501—were seen as part of “civilized inheritance” in the same vein as antiquities or ancient manuscripts. These books therefore became items that needed to be preserved in order to pass on to future generations. The need to identify what was important enough to be passed on, combined with the proliferation of bibliographies and literature about books themselves, resulted in the formation of hierarchical canons of books. For instance, editio princeps, major illustrated works, and works of important printers such as Aldus Manutius were always in demand and expensive; then came lesser literature; then came publications that were seen as having no value and were often relegated to waste paper.

Andreas Vesalius's 1543 atlas De humani corporis fabrica provides insight into how these various factors impacted rare medical books in particular. The Fabrica is regarded as one of the seminal texts in the history of anatomy and is a highlight of many medical rare book collections. It was, however, not always considered a rare book desirable for its prestige—it was a practical text for working physicians. In their survey of known copies of the 1543 and 1555 editions, historian Dániel Margócsy and his team found evidence that early owners were actively engaged with the text [7]. Annotators put references to other anatomists both ancient and contemporary in the margins to note points of agreement and disagreement, summarized Vesalius's arguments, and of course left their share of readers' marks. While the Fabrica was certainly valuable enough to serve as a particularly noteworthy gift among educated physicians; medical practitioners still found its content useful enough to incorporate it into their working libraries.

In Margócsy et.al's analysis, the Fabrica's ascension from expensive but useful anatomical text to rare collector's item can be attributed to two key factors: significant changes in medical practice and research in the years surrounding 1800 and the rise of book collecting among North American physicians. As histology and comparative anatomy gained prominence and the rise of chromolithography allowed anatomy texts to be printed in color, older texts such as the Fabrica were no longer relevant as practical guides to the study of anatomy. This relegated the Fabrica to the stuff of medical history, and its price dropped significantly during the late eighteenth and nineteenth centuries.

The collecting practices of North American physicians then turned the Fabrica into a collector's item. In the late nineteenth century, it was fairly common for American physicians to spend time training in Europe, where they could acquire copies of the Fabrica. Over time, this gave rise to the so-called Cult of Vesalius, where Vesalius' works were particularly prized additions to medical collections. The two most important figures in this development were arguably William Osler and Harvey Cushing, both of whom were monumental figures in the development of North American medicine at Johns Hopkins and Yale. Osler was particularly enamored with Vesalius. As noted by Margócsy, Osler owned seven 1543 and two 1555 Fabricas over the course of his career, and strategically donated them to North American medical libraries because of his belief that “we cannot have too many copies in America & no medical library is complete without one.” The case of the Fabrica proves McKitterick's point nicely: rare books are defined by the collectors who decide what is important and desirable. While the Fabrica is not a particularly scarce book—we know of more than one hundred copies in institutional libraries, and there are certainly more in private collections—it is held in such high regard by doctors and historians of medicine that it is practically an essential addition to any collection of rare medical texts.

The tastes and collecting practices of twentieth-century physicians have certainly influenced the development of the rare book collections at Becker Library, as an examination of its history shows. The medical library of the Washington University in St. Louis School of Medicine was first mentioned in the school's 1902-1903 annual announcement. In 1910, the medical faculty received $15,000 to support the library, adequate space to carry out all essential library tasks, and a promise for an annual budget. This sudden largesse prompted a committee of medical department heads to develop a strategic plan for the library's future. At this juncture, as noted by George Dock in his summary of the medical library's collecting history, there was to be no “effort at building a book museum, nor buy antiques out of the budget, but to aim at getting through gift items that formed landmarks in the history of medicine [3].”

The library's first major acquisition of rare books occurred in 1912, when the collection of the German medical historian and bibliophile Julius Pagel's personal collection was offered for sale. Pagel received his medical degree from the University of Berlin in 1876, but his true passion was the literary, human, and sociological aspects of the history of medicine, rather than clinical practice. His fascination with medical history was reflected in his book collection, which consisted of some 2500 volumes and included pre-1600 monographs by important medical authors including Dioscorides and Avicenna, seventeenth and eighteenth century works such as those authored by Morgagni and Malpighi; and a number of works on medical biography, bibliography, and art.

At the time of Pagel's death, there were several bibliophiles on the medical school's library committee, the most significant of whom were George Dock and Frank J. Lutz. Dock (1860-1951) received his medical degree from the University in Pennsylvania in 1884 and came to Washington University in 1910, where he served as both Dean of the Medical School and Professor of Medicine before leaving for California in 1922. He was a great supporter of medical libraries, and served as President of the Medical Library Association from 1906 to 1909. He was not only interested in current medical literature, but appreciated historical texts as well – an interest that might have been encouraged by the great medical collector William Osler, who he met at the University of Pennsylvania. Like his mentor Osler, Dock assembled an impressive rare book collection. Most of his rare volumes were eventually donated to the Library of the Los Angeles County Medical Association, but he also gave several volumes to Washington University during his tenure there.

The other prominent bibliophile on the library committee was Frank J. Lutz (1855-1916). Lutz earned his medical degree from the St. Louis Medical College in 1876 and later taught at the Washington University Medical Department. Like Dock, Lutz was both an avid supporter of medical libraries and a collector of historical texts. In December 1914, his historical and bibliographic interests came together at an exhibit documenting the history of medicine held at the Jefferson Memorial (now the Missouri History Museum), which featured a number of early modern books from Lutz's personal collection.

Given the bibliographic leanings of these two gentlemen, it is no surprise that they were interested in Pagel's collection. Funds for the purchase were provided by the prominent St. Louis philanthropist Christine Blair Graham thanks to the efforts of yet another library committee member, Dr. W.E. Fischel. The Pagel collection came to Washington University in 1914, where it became the bedrock of the medical library's rare book holdings.

These holdings grew considerably over the next century. One of the most substantial donations was the ophthalmological collection of Bernard Becker (1920-2013), who served as head of the medical school's Department of Ophthalmology and Visual Sciences from 1953-1988. In his oral history conducted in 1990, Dr. Becker mentioned his longstanding interest in books. When he traveled, he would often purchase interesting items from old book stores and have them shipped home; these purchases were then supplemented with gifts from his father-in-law, who was interested in “collectors' items,” and he eventually amassed a collection of some 600 volumes. In the introduction to the annotated Becker catalogue, he stated that:

**As the collection grew, I began to feel rather selfish about the possessions of these rare and sometimes unique books and developed the need to share the experiences they offered with others. It has always been my firm conviction that books, like ideas, do not belong to individuals; they must be available to everyone [14].**

This spirit of generosity led him to donate the collection to the library in 1975, the largest acquisition of rare books since the purchase of the Pagel collection.

Another major twentieth century donation was the CID-Max A. Goldstein Collection in Speech and Hearing. This collection was assembled by Max Aaron Goldstein (1870-1941), a St. Louis native who graduated from the Missouri Medical College in 1892. Like many other physicians of this time period, he then spent two years training in Europe at the Vienna Polyclinic, where he studied with the prominent otologist Adam Politzer. He later founded the Central Institute for the Deaf in St. Louis, an institution where teachers and otologists could collaborate on deaf education, in 1914.

Goldstein was also a passionate book collector, and from 1922 to 1939 he assembled a fine collection of rare texts dating mainly from the sixteenth through nineteenth centuries. A look through his correspondence reveals that he was a savvy collector who maintained an active relationship with numerous booksellers in both Europe and North America, including Maggs Brothers, a London bookseller that is still in operation today; and Wilfrid Voynich, the renowned antiquarian bookseller who purchased the eponymous Voynich Manuscript. His collection was housed at the Central Institute for the Deaf until it came to Becker Library in 1977.

The final collector discussed here is H. Richard Tyler, a Washington University alumnus who went on to teach at Harvard Medical School. Tyler credited a physician he worked with as an intern at Brigham for sparking his initial interest in collecting medical history. He decided to focus on the history of neurology because, as he said in his oral history conducted in 2013 with the American Academy of Neurology, “[The book dealers] knew what was important in books and what the price of books were and what was rare, but they didn't know necessarily all the people who were important. So there was a chance of doing something [11].” He became a prolific collector, amassing enough books that they eventually took over a considerable amount of real estate in his house.

Tyler was also not necessarily collecting books just for the sake of collecting books. He was also at least somewhat aware of rare books as markers of prestige. In the same interview referenced above, he stated that:

**I…was not oblivious to the fact that Cushing's major contribution in my mind was the donation of his books to Yale. And the Yale Rare Book Library has Cushing's collection and it will stand the test of time…They know he was the father of neurosurgery, but they will never forget the fact that, if you want to see Vesalius, you will have to go to Cushing's collection. [11]**

From this point of view, book collections are not just something assembled out of personal enjoyment. They are also a matter of personal legacy, of passing the knowledge that the donor deems important on to future generations, and the books will stand as a monument to that particular collector's tastes and interests even after they themselves pass away.

When we look at these figures who shaped Becker Library's collections, we can observe several commonalities. All of them were male doctors of European descent who were highly educated, able to travel to Europe, and had enough disposable income that they could purchase expensive texts. These broad similarities are reflected in the collections they assembled. There are several cases of overlap within the library's collections, and Becker holds multiple copies of Vesalius, Galen, Hippocrates, and other “classics.” While each collector tended to favor the specific medical subjects that interested them, if we look at the books as a whole, they tell a story focused almost exclusively on the development of medicine in Europe and North America from the late 1400s onward, and texts representing other medical traditions are extremely few and far between. Nor is Becker Library alone in that regard. The rare books canon that is preserved in most North American libraries is primarily a story of medical development in the global West.

There is nothing wrong with having an interest in and wanting to collect materials that reflect the academic and medical heritage one was trained in. It is understandable that Bernard Becker wanted to collect the classics of ophthalmology, H. Richard Tyler focused on neurology, and so forth. There are other factors at play as well. One of them is the availability of books—during the interwar period in particular, institutional libraries sold items to booksellers in order to raise funds, and the collections of these libraries were focused on European heritage. Moreover, while doctors were definitely able to read and speak at least German in addition to English, and had some knowledge of Latin and Greek, they were less likely to be proficient in non-European languages. They therefore might have preferred to collect items they were able to read themselves.

But special collections are places of power, and the items within them occupy a privileged position. They are not held in the open stacks, where anyone can browse them without supervision, they are kept in special secured areas and can only be viewed by filing a request. Furthermore, as special collections librarian Jesse Erickson has noted, special collections architecture often sets out to evoke the architecture of a European gentleman's ideal study [4]. When the majority of materials in special collections are Eurocentric and are presented in a setting that echoes ideas of European power and respectability, it can send a message—albeit unintentionally—that European texts are the most significant and worthy of study.

Over the past several years, more and more special collections divisions in North America are looking to expand their programming and collections beyond a Eurocentric focus. This goal is shared by many medical special collections. As institutions begin to reexamine their holdings, it is important to bear in mind that building collections that reflect a global history, rather than a Eurocentric one, is an ongoing process that cannot be accomplished overnight. Acquisition budgets and storage capacity will always be important considerations for libraries as they evaluate their collecting priorities, and collecting a few token works is not an effective approach. But the history of medicine is inherently international, which can provide an opportunity for stewards of medical collections to find areas that can serve as natural expansion points within a collecting policy. To name a few: the Greco-Roman philosophies of Hippocrates and Galen were preserved by Arabic physicians during the medieval period, whose writings then became standard medical texts in Europe; Dutch traders learned about acupuncture during their time at the island of Dejima in Nagasaki, Japan while Japanese physicians learned about western medicine and published Japanese translations of and commentaries on Western scientific works; and European herbals incorporated descriptions of medicinal plants from the Americas.

In the case of Becker Library, we have opted to begin expanding our collections based on our existing strengths. Becker's ophthalmology collection already contains most of the key Western ophthalmological texts; therefore, expanding our holdings in that subject to encompass ophthalmological materials from other medical traditions is a logical next step in building that collection. Our holdings in the history of anatomy are also very strong, and we are working to acquire works that complement them. For example, over the past year, we have bought several Japanese rangaku texts whose Dutch counterparts are held within our collections. These provide us with an easy starting point to begin talking about the transmission of medical knowledge, and also serve as a bridge for acquiring and highlighting original Japanese texts. While we are extremely fortunate that our initial donors were such prolific and discerning collectors, we now hope to plant the seeds for a collection that can tell a global history of medicine, rather than a history of medicine that focuses almost exclusively on the European perspective.
